# Fluoxetine does not influence response to continuous theta burst stimulation in human motor cortex

**DOI:** 10.1002/npr2.12493

**Published:** 2024-11-07

**Authors:** Duncan K. Austin, Lourenço M. D. Amador, Lucia M. Li, Simon J. Little, John C. Rothwell

**Affiliations:** ^1^ University College London London UK; ^2^ Monash University Melbourne Australia; ^3^ University of Melbourne Melbourne Australia; ^4^ Imperial College London London UK; ^5^ Department of Neurology University of California San Francisco USA

**Keywords:** antidepressants: basic, psychopharmacology: basic

## Abstract

**Aim:**

Selective serotonin reuptake inhibitors are thought to exert a clinical effect through various mechanisms, including through alteration in synaptic plasticity. Repetitive transcranial magnetic stimulation can induce temporary changes in synaptic excitability in cerebral cortex that resemble long‐term potentiation and long‐term depression that serve as a measure of synaptic plasticity in vivo. A version of repetitive transcranial magnetic stimulation called continuous theta burst stimulation can induce inhibition of cortical excitability that can be measured through a motor evoked potential. Previous work has suggested that this response can be modulated by administration of selective serotonin reuptake inhibitors.

**Method:**

Thirty‐one healthy volunteers received both fluoxetine 20 mg and placebo in randomly ordered sessions, followed by spaced continuous theta burst stimulation to motor cortex. Changes in Motor Evoked Potentials were then recorded over 60 min.

**Results:**

The response to spaced continuous theta burst stimulation did not differ significantly between fluoxetine and placebo sessions. Spaced continuous theta burst stimulation produced a paradoxical excitatory response in an unexpected number of participants.

**Conclusion:**

A single dose of fluoxetine 20 mg does not influence the response to continuous theta burst stimulation. Previous results suggesting an effect of selective serotonin reuptake inhibitors on inhibitory non‐invasive brain stimulation protocols may be due to insufficiently large sample sizes.

## Abstract 2

cTBS can produce both inhibition and paradoxical facilitation of cortical MEPs. Contrary to the impact of other SSRIs on alternative forms of non‐invasive brain stimulation, fluoxetine 20 mg did not modulate the effect of cTBS.

## Graphical abstract

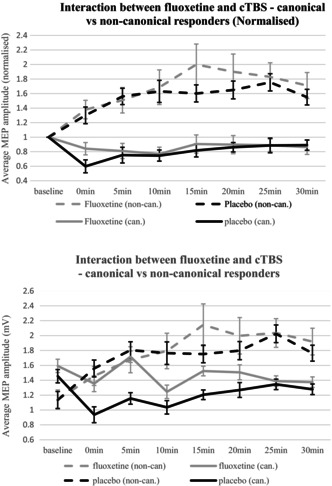

## 1 INTRODUCTION

Fluoxetine, a selective serotonin reuptake inhibitor (SSRI), has been licensed since the 1980s for the treatment of depression. Although its effect on serotonin reuptake is seen immediately *in vitro,* ^1^ clinical anti‐depressant effects are typically only seen several weeks later^2^ and it has been suggested the drug may be exerting its clinical effect through alterations in neural plasticity.^3^ Animal studies have found that serotonin increases long‐term potentiation (LTP) and inhibits long‐term depression (LTD) *in vitro,* ^4^ and non‐invasive brain stimulation (NIBS) using both transcranial direct current stimulation (TDCS)^5^ and paired afferent stimulation (PAS)^6^ to induce changes in synaptic plasticity have found in vivo changes in cortical excitability that mirror LTD and LTP following administration of a single dose of SSRI. Various NIBS protocols exist using various forms of repetitive transcranial stimuli to induce short‐lasting changes in cortical excitability in an LTP‐like or LTD‐like fashion, and have become leading tools for measuring plasticity in human cerebral cortex in vivo.^7^ However, no NIBS study to date has explored the effect of fluoxetine, despite this agent being among the most widely prescribed antidepressants and despite it being considered a leading candidate for pharmacological enhancement of neural plasticity.^8^, ^9^, ^10^

Modern non‐invasive brain stimulation paradigms using patterned repetitive stimulation protocols such as theta‐burst stimulation (TBS)^11^ and have demonstrated superior reproducibility and reduced intra‐subject variation.^12^ Typically, an intermittent pattern of theta‐burst stimulation (iTBS) produces LTP‐like changes, whereas continuous theta‐burst stimulation (cTBS) produces LTD‐like changes.^13^ Recent studies^14^ have found this effect to be more reliable and more sustained when applied in a spaced protocol with a 10‐min inter‐train interval, representing a promising tool for probing the actions of psychopharmacological agents *in vivo*.

### 1.1 Hypotheses

It was postulated that oral administration of a single dose of fluoxetine 20 mg would enhance the inhibitory effect of a spaced cTBS applied to motor cortex, measured as a reduction in the mean amplitude of the Motor Evoked Potential (MEP) over 30 min.

## 2 METHODOLOGY

### 2.1 Power calculation

Based on the laboratory data^15^ suggesting an expected standard deviation (0.2) and intraclass correlation (0.5) of the primary outcome measure (average change in MEP amplitude over 30 min following theta burst stimulation), and anticipating a modest effect size of fluoxetine 20 mg (0.2), a target sample of 31 subjects was deemed sufficient to detect a significant difference with 80% power.

### 2.2 Participants

31 healthy volunteers (average age 26.3 years. ±5.6, range 20–47; 30 right‐handed (RH) 1 left‐handed (LH), 20 male (M) 11 female (F)) were recruited and screened for any chronic neurological conditions and for any contra‐indication to receiving either non‐invasive brain stimulation or fluoxetine. Subjects with a history of significant mood disorder or epilepsy, or currently taking antidepressant medication, were excluded. The study was approved by the local Research Ethics Committee at University College London and performed in accordance with the principles of the Declaration of Helsinki. All subjects gave informed written consent and completed both sessions.

### 2.3 Experimental procedure

Subjects were given either fluoxetine 20 mg in liquid form or placebo (mint syrup) under double‐blind conditions and then asked to return to the laboratory for testing 6 h later to coincide with peak plasma fluoxetine levels^16^ following oral administration. Consequently, all testing took place in the afternoon or early evening. All subjects returned for a second testing session a minimum of 72 h later (average interval 23 days, range 3–65), where they received the other alternative of either fluoxetine or placebo (order was randomly allocated for each subject by a non‐blinded investigator). The second session was otherwise identical to the first. After the second session, the subjects were given a forced‐choice question asking them whether they thought that they received fluoxetine or placebo in each session.

### 2.4 Electromyography

Surface electromyography (EMG) was used to record the MEP from the first dorsal interosseous (FDI) muscle of the dominant hand using Ag/AgCL electrodes (Ambu, Ballerup, Denmark) in a belly‐tendon montage. Skin was prepared by cleaning with alcohol and lightly abrading with NuPrep paste. A ground electrode was placed on the radial styloid process. EMG signal was sampled at 5 kHz (CED 1401, Cambridge Electronic Design, Cambridge, UK), amplified 1000x (Digitimer D360™, Welwyn Garden City, Herts, UK), band‐pass filtered (30–1000 Hz), and stored for offline analysis (Signal™ software, Cambridge Electronic Design, Cambridge).

### 2.5 Transcranial magnetic stimulation

Single pulses of TMS were delivered using a Magstim™ 200^2^ stimulator (Magstim Co., Whitland, Dyfeld, UK) and figure‐of‐eight 70 mm internal diameter Alpha coil (Magstim Co., Whitland, Dyfeld, UK), held tangentially to the scalp with the handle positioned 45° posterolateral to induce a posterior–anterior current across motor cortex. The optimal coil position for evoking MEPs in the FDI muscle at rest, defined as the position which yielded the largest and most consistent MEP on FDI EMG, was located using systematic adjustment of coil position relative to surface landmarks and fixed using Brainsight™ neuronavigation system (Rogue Resolutions Inc., Cardiff, UK) without the incorporation of neuroanatomical imaging. MEPs collected when the coil was more than 1 mm or 3° off target in any plane were discarded and repeated.

Resting motor threshold (RMT) was defined as the minimum stimulus intensity (measured as percentage of maximum stimulator output) required to evoke an MEP with a peak‐to‐peak amplitude larger than 50 μV in at least 5 of 10 trials. Baseline corticospinal excitability was measured by recording a block of 30 MEPs at the stimulus intensity producing an average MEP closest to 1 mV in amplitude. Trials contaminated with pre‐stimulus muscle activity were discarded and repeated. Following theta‐burst stimulation, further blocks of 30 MEPs were recorded at 5‐min time intervals up to 30 min. Peak‐to‐peak amplitude was measured using an in‐house developed script and averaged across all MEPs from that time point. Plasticity was quantified as the change in mean MEP amplitude from baseline post cTBS at respective time points.

### 2.6 Continuous theta burst stimulation

The cTBS protocol consisted of 600 pulses of TMS delivered in triplets at 50 Hz, repeated at 5 Hz for a total of 40 s.^11^ A Magstim Rapid™ stimulator connected to an air‐cooled figure‐of‐eight coil (Magstim Company, Dyfed, UK) was used to apply cTBS with a biphasic pulse waveform to the optimal scalp position for evoking responses in the FDI. A paired cTBS paradigm was used of two cTBS trains (600 pulses each) separated by a 10 min interval.^14^ Between cTBS trains, participants were asked to relax quietly and refrain from muscle contraction of the upper limb. The intensity of stimulation was set to 70% of the RMT intensity.^14^, ^17^

### 2.7 Paired‐pulse stimulation

After baseline MEPs were recorded and prior to administration of cTBS, a random‐sequence paired pulse protocol probing intracortical circuits was delivered. Short intracortical inhibition (SICI) and intracortical facilitation (ICF) were assessed by randomly cycling between a condition where a single monophasic test pulse was delivered in isolation at a preset intensity intended to invoke a 1 mV amplitude MEP, and 4 other conditions where a 1 mV intensity pulse was preceded by a monophasic subthreshold (70% RMT) conditioning pulse at an interval of 2, 3, 10, or 15 ms.^18^ SICI, felt to represent the action of GABA_A_ergic cortical interneurons, and ICF, felt to represent glutamatergic excitatory cortical interneurons,^19^ were assessed as respective increase or decrease in the conditioned pulse MEP amplitude compared to the test pulse MEP amplitude. Ten trials of each condition (10× test pulse alone, 10 test pulse conditioned at 2 ms, 10× test pulse conditioned at 3 ms, 10× test pulse conditioned at 10 ms, 10× test pulse conditioned at 15 ms – total 50 trials) were performed in each session in computer‐generated random order at a frequency of 0.2 Hz.

In a separate paired‐pulse protocol, long intercortical inhibition (LICI) was assessed. LICI is felt to represent GABA_B_ergic activity^20^ in more distant or recurrent neurons.^21^ A 1 mV monophasic test stimulus was utilized, delivered alone in 15 trials of 30 trials and preceded at an interval of 150 ms by another 1 mV intensity conditioning stimulus in the other 15 trials. 30 MEPs were collected in a computer‐generated random order at a frequency of 0.2 Hz. As with SICI, LICI was defined as a decrease in the mean amplitude of the conditioned MEP compared to the test stimulus MEP.

## 3 STATISTICAL ANALYSIS

All statistical analysis was performed using SPSS (Version 22.0., 2013, Armonk, NY: IBM Corp). An individual's MEP amplitudes at each time point was averaged to produce a single value, and then normalized to baseline for each individual to correct for baseline variation. Data were transformed where to necessary to meet the assumptions of the parametric model.

A two‐way repeat‐measures ANOVA was performed with factors SESSION (fluoxetine/placebo) and TIME (baseline, 0, 5, 10, 15, 20, 25, and 30 m). A paired sample t‐test was used to compare RMT between groups and to compare the effects of paired pulse data. The proportion of correct answers on the forced‐choice questionnaire was analyzed using a Chi‐Square test.

## 4 RESULTS

### 4.1 Baseline parameters

RMT did not differ between sessions (45.0% vs. 45.7%: t(30) = 0.706, *p* = 0.485). Baseline MEP amplitude did not differ across sessions (fluoxetine 1.30 mV SD 0.54; placebo 1.25 mV SD 0.68: t(30) = 0.517, *p* = 0.609).

### 4.2 Intracortical excitability

Comparing paired‐pulsed stimulation (Table 1), there was no difference in mean SICI (*p* = 0.845), LICI (*p* = 0.11) and SICF (*p* = 0.455; Table 1).

**TABLE 1:** Pair pulsed change in MEP as a proportion of unconditioned MEP.

*ISI*	*SICI*	*ICF*	*LICI*
Placebo	0.511	1.279	0.288
Fluoxetine	0.521	1.382	0.398
Sig. (*p*‐value)	0.845^a^	0.455	0.110

^a^ Wilcoxon Signed Ranks Test—data could not be transformed to meet the assumptions of the parametric model.

### 4.3 Response to cTBS

**FIGURE 1:**
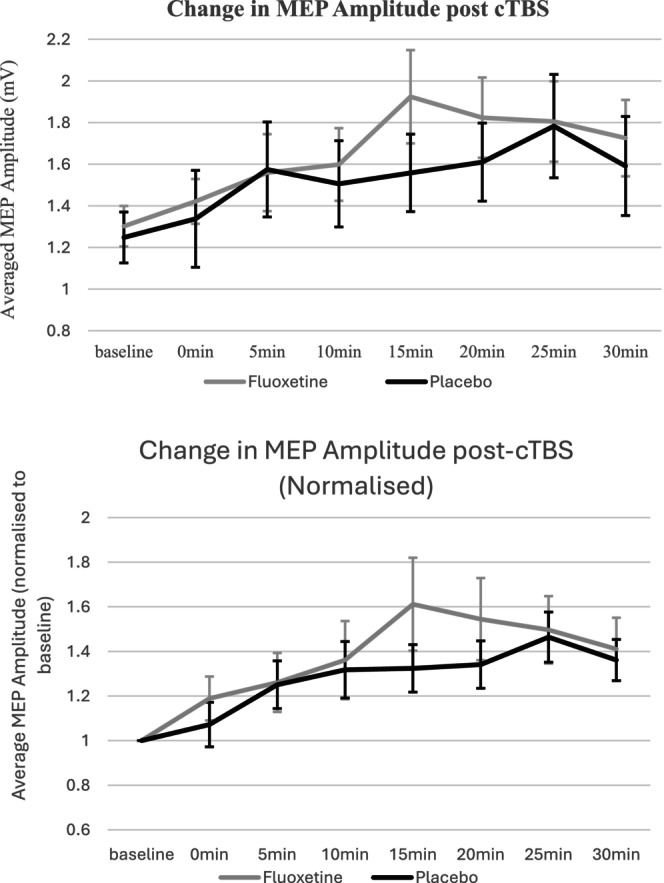
Absolute and relative change in MEP amplitude post cTBS – fluoxetine versus placebo session – bar lines show standard error.

There was a significant effect of TIME post cTBS on MEP amplitude (F^(3.6108.3)^ = 5.888, *p* < 0.001), but no effect of SESSION (*F*(1, 30) = 2.14, *p* = 0.154) or SESSION × TIME interaction (*F*(4.2, 126.9) = 0.80, *p* = 0.533), suggesting that cTBS was influencing MEP amplitude, but that fluoxetine was not affecting MEPs or modulating the effect of cTBS (Figure 1).

### 4.4 Non‐canonical response to cTBS

There was a high proportion of subjects showing an atypical (non‐canonical) response to cTBS in our cohort. Individual response to cTBS was predominantly facilitatory in both sessions, with only 11 of 31 subjects showing a net inhibitory response (mean MEP < 100% baseline) to cTBS in the placebo session and 10 showing inhibition in the fluoxetine session. Response was however highly concordant, with 26 out of 31 (83.9%) showing the same response (facilitation/inhibition) in both sessions. Despite differing pharmacological conditions, intraclass correlation between the two recordings was good (ICC = 0.592 CI [0.154, 0.803] *p* = 0.008). ICC improved yet further when data from the first 10 min post stimulation were used, improving to 0.651 (CI [0.277–0.832], *p* = 0.003).

**FIGURE 2:**
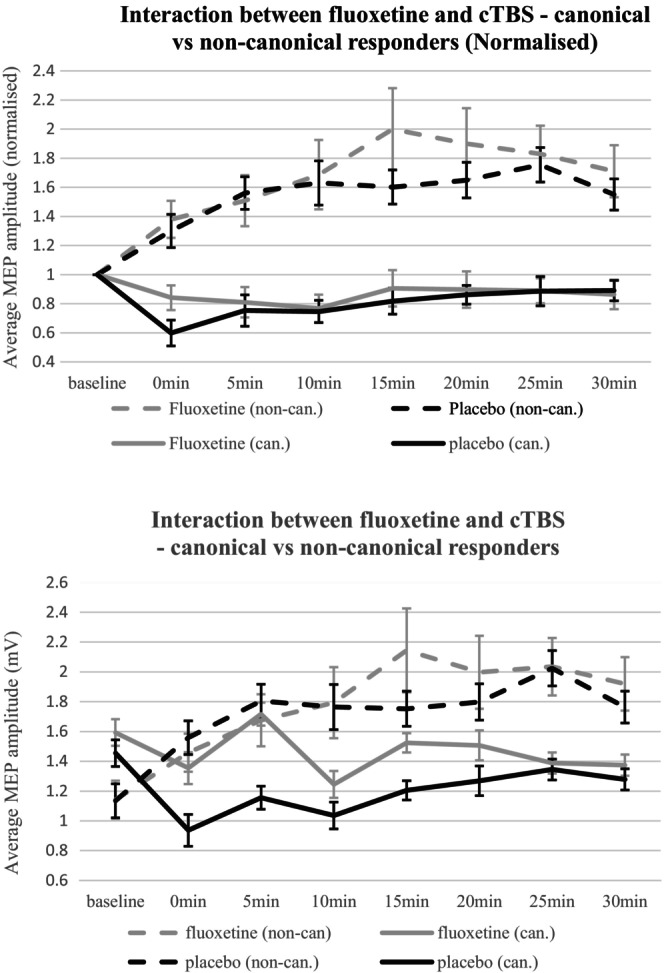
Absolute and relative change in MEP amplitude post cTBS in canonical and non‐canonical responders—fluoxetine vs. placebo.

Given that the high proportion of non‐canonical responders in this sample, we repeated the ANOVA using RESPONDER as a covariate to see if this could better capture any effect of fluoxetine. Using response across 30 min in the placebo session as the determinant of whether they were classified as RESPONDER or NON‐RESPONDER, this factor was added as a covariate in the ANOVA but did not affect the analysis, with no SESSION*TIME*RESPONDER interaction (*F*(4.2, 111.1) = 0.675, *p* = 0.604). Thus, the effect of fluoxetine on response to cTBS did not differ between canonical and non‐canonical responders (Figure 2).

## 5 DISCUSSION

Resting motor threshold also did not differ between sessions in our subjects, which is in keeping with the existing human data, which do not support an effect of fluoxetine on RMT.^22^, ^23^ Other studies have also found no effect of SSRIs on baseline corticospinal excitability, including citalopram,^6^, ^24^ sertraline,^25^ mirtazapine,^26^ or paroxetine.^27^ Other studies with citalopram have produced different findings, with Robol et al.^28^ finding an increase in motor threshold in healthy subjects given citalopram, and a similar increase in RMT with citalopram was found in the unaffected hemisphere of stroke patients.^29^

There is thus a significant divergence in the published data as to the effects of SSRIs on corticospinal excitability, with only citalopram demonstrating an effect, decreasing cortical excitability and increasing in intracortical inhibition.^5^ Fluoxetine, paroxetine, and sertraline all exert a weak effect on norepinephrine, acetylcholine, and dopamine reuptake mechanisms,^30^ whereas citalopram is a much more selective 5‐HT reuptake inhibitor, which might conceivably be responsible for the discordant results.

These data also suggest that a single dose of fluoxetine 20 mg does not influence the effect of cTBS on human motor cortex. This is at odds with the significant influence of other SSRIs on alternative non‐invasive brain stimulation methodologies in previous studies on healthy volunteers. This may reflect an aspect of either the investigation product or of the brain stimulation protocol chosen. However, McDonnell et al.^22^ found no effect of fluoxetine on motor learning in a thumb‐abduction task, a process which has been shown to be dependent on LTP plasticity in motor cortex, and which produces an LTP‐like increase in MEPs which can be reversed with cTBS.^31^ Thus, there is some additional indirect evidence that fluoxetine does not influence LTP in human motor cortex (and that if it did we might expect the effect to be detectable using cTBS).

The lack of effect on either ICF or ICI is at odds with data in stroke patients,^10^ which found both an increase in ICF and a decrease in ICI with fluoxetine, although this was after chronic administration of the drug. Other small studies have found evidence for increased ICF with sertraline^25^ and paroxetine,^27^ whereas others have found an increase in ICI,^28^ or no effect at all.^26^ Overall, the previous data from small studies have been heterogeneous and conflicting, whereas the present data provide more convincing evidence for lack of effect.

The present study has failed to replicate an important finding of Nitsche et al.^5^ and Melo et al.^32^ In their work, the inhibitory (LTD‐like) effect of TDCS on MEPs was converted into a facilitatory (LTP‐like) effect by citalopram. Serotonin is felt to influence LTD through activation of 5HT _(2)_ receptors,^33^ and since fluoxetine and citalopram differ in their affinity for this receptor,^34^ this pharmacological issue could also potentially explain their different interactions.

Cathodal TDCS and cTBS have different mechanisms of action, as TDCS modulates neuronal membrane potential through constant polarizing weak currents rather than patterned stimulation. Furthermore, TDCS has a less defined spatial resolution and may activate pathways outside of primary motor cortex. Nevertheless, methodologies both produce a similar impact on motor cortex plasticity when measured using TMS, and there is no a priori reason why TDCS should capture an effect of SSRIs not seen with TBS. The reliability of responses to PAS^35^ and TDCS^36^ have not been shown to be superior to TBS, with a recent critical review citing broadly similar responder rates across the three modalities,^37^ suggesting that findings with citalopram by Nitsche et al. and Melo et al. cannot necessarily be attributed to the technical superiority of their choice of NIBS. Equally, the sample size in both studies was only 12 subjects, suggesting they may have been significantly underpowered given the ICC of TDCS is broadly comparable to cTBS.^36^

A puzzling factor in our own results was the fact that a majority of subjects in this study demonstrated a non‐canonical response to cTBS, whereas typically the largest proportion of subjects shows an inhibitory effect.^38^ Such a paradoxical response to cTBS was previously demonstrated by Gamboa et al.^39^ when cTBS was preceded by a ‘priming’ run of cTBS. However, in our protocol there was a 10‐min inter‐train rest interval based on the results of Goldsworthy et al.,^14^ whereas in their study the two cTBS protocols ran continuously back‐to‐back.

The paradoxical effect in the current study was seen in both sessions and therefore seems unrelated to the ingestion of fluoxetine, but rather to have been driven by an unexpected large number of subjects showing a consistent non‐canonical response across both experimental conditions. The consistency of response across the two sessions was high (Intraclass Correlation 0‐59‐0.65) implying that a genuine physiological response has been captured and not merely ‘noise’ arising out of the baseline variability in the MEP. It has previously been noted^12^ that response to theta burst stimulation is fairly consistent within subjects, implying that when experimental parameters are kept constant, non‐canonical response is determined by fixed intrinsic biological factors. Cheeran et al.^40^ found that BDNF polymorphisms influenced i/cTBS response, with Val66Met carriers significantly differing from that of Val66Val subjects, and the authors suggested that this was due to the influence of BDNF on synaptic predisposition to undergo LTP/LTD.

In clinical scenarios, pharmacologically‐driven improvements in motor function have generally only been seen when in combination with rehabilitative motor learning therapy,^41^ although short‐term changes detected with NIBS are thought to be the substrate that facilitates subsequent functional improvement.^42^ SSRI typically take several weeks to manifest a clinical response, and chronic administration of SSRIs is known to enhance the cAMP‐dependent phosphorylation of proteins such as BDNF.^43^ A single dose of fluoxetine in this study may therefore have been insufficient to modulate the BDNF‐driven effect of cTBS. Nevertheless, it is worth repeating that single doses of citalopram have demonstrated an effect with other forms of NIBS.^5^, ^6^, ^32^

## 6 CONCLUSIONS

cTBS produced an unexpected non‐canonical response in a majority of subjects, but fluoxetine had no effect on cortical excitability or cTBS‐induced plasticity. Although other SSRIs, particularly citalopram, have been found to demonstrate metaplastic effects, the existing data do not support a role for fluoxetine in modulating plasticity in human motor cortex.

## AUTHOR CONTRIBUTIONS

DKA, LML, SJL and JCR designed the study. DKA and LMDA collected and analyzed the data. DKA prepared the manuscript and LMDA, LML, SJL and JCR reviewed and contributed to the final submission.

## FUNDING INFORMATION

The work presented in this paper was supported by funding from The Stroke Association.

## CONFLICT OF INTEREST STATEMENT

The authors of this work declare that they have no competing interests to disclose.

## ETHICS STATEMENT

Approval of the Research Protocol by an Institutional Reviewer Board: The research protocol was approved by the UCL Research Ethics Committee.

Informed Consent: Written informed consent was obtained from all participants.

Registry and the Registration No. of the Study/Trial: N/A.

Animal Studies: N/A.

## Data Availability

The raw data collected during this study have been deposited with the Open Science Framework and are freely available for inspection and study (Austin, D. (2024, August 27). fluoxetine does not influence response to continuous theta burst stimulation in human motor cortex. Retrieved from osf.io/yze8b).
